# Clinical Utility of SARS-CoV-2 Antibody Titers in the Management of Patients With Long COVID Infected With the Omicron Variant

**DOI:** 10.3389/bjbs.2026.16255

**Published:** 2026-04-22

**Authors:** Marina Kawaguchi, Yasue Sakurada, Kazuki Tokumasu, Yuki Otsuka, Yasuhiro Nakano, Yui Matsuda, Hiroyuki Honda, Daisuke Omura, Nobuyoshi Matsuki, Masanori Furukawa, Akihito Higashikage, Fumio Otsuka

**Affiliations:** 1 Department of General Medicine, Okayama University Graduate School of Medicine, Dentistry and Pharmaceutical Sciences, Okayama, Japan; 2 Department of Laboratory Medicine, Okayama University Hospital, Okayama, Japan

**Keywords:** brain fog, COVID-19, long COVID, Omicron variants, SARS-CoV-2 antibodies

## Abstract

**Background:**

Long COVID (LC) presents persistent symptoms that pose a major clinical challenge. Identification of reliable biomarkers to evaluate LC pathophysiology is needed.

**Objectives:**

To investigate whether serum S- and N-antibody titers against SARS-CoV-2 spike and nucleocapsid proteins reflect the clinical features of LC.

**Methods:**

This retrospective observational study included patients diagnosed with Omicron variant-related LC who attended a post-COVID-19 outpatient clinic between July 2023 and November 2024 and provided informed consent for antibody testing.

**Results:**

Among 275 patients (129 men and 146 women), 57 (21%) were unvaccinated. Median S- and N-antibody titers in vaccinated versus unvaccinated patients were 20,963 U/mL and 24.8 cut-off index (COI) versus 24 U/mL and 44.5 COI, respectively. S-antibody titers were associated with the number of vaccine doses received, whereas N-antibody titers correlated with disease severity during the acute phase of COVID-19 infection, with females having higher titers by multivariable analysis. N-antibody titers in unvaccinated patients with LC were negatively correlated with time interval from infection to clinic visit, with an estimated daily decline of 0.34% in measured N-antibody levels. Patients with LC having memory impairment had low S-antibody titers by multivariable logistic regression analysis, and low S-antibody levels were associated with reduced quality of life (QOL). Additionally, N-antibody titers positively correlated with lymphocyte counts and immunoglobulin levels.

**Conclusion:**

Serum N-antibody titers reflect immune responses to COVID-19, although they are affected by gender differences and interval between infection and evaluation. Lower S-antibody titers were associated with brain fog symptoms and reduced QOL in patients with LC.

## Introduction

Coronavirus disease 2019 (COVID-19) results in a wide range of symptoms not only during the acute phase of severe acute respiratory syndrome coronavirus 2 (SARS-CoV-2) infection but also several months after infection, a condition referred to as post-COVID-19 condition, post-acute sequelae of COVID-19, or long COVID [1, 2]. Long COVID is defined as a condition characterized by a set of symptoms that typically arise within 3 months of COVID-19 onset, persist for at least 2 months, and cannot be explained by an alternative diagnosis [3]. Clinical manifestations of long COVID encompass a broad range of symptoms including fatigue, dyspnea, and cognitive impairment. Although some aspects of the pathophysiology of long COVID have been elucidated, they remain complex and are thought to involve mechanisms such as microthrombi formation, inflammation, autoimmunity, viral persistence, neurological injury, and vascular dysfunction [4]. This complexity underscores the need for a multidisciplinary approach rather than relying on a single therapeutic strategy. Long COVID exhibits a wide spectrum of severity, including mild and severe symptoms, substantially affecting daily activities and work [5].

Long COVID encompasses a broad spectrum of underlying mechanisms and clinical manifestations [6, 7]. Epidemiological evidence indicates that the overall risk of developing long COVID has declined in Europe and Japan from the early pandemic strains to the phase of infection with the Omicron variant, which is partly attributable to widespread vaccination [8–11]. Nevertheless, 4%–10% of individuals infected with COVID-19 continue to develop long COVID [8, 10, 12], indicating that it remains a substantial public health concern. Furthermore, in Japan, the risk of cognitive symptoms, such as brain fog, is relatively higher during the Omicron phase than that during infection with earlier variants [13].

We reported that approximately 6 months represents a potential timepoint for distinguishing self-limited from prolonged courses of long COVID among Japanese patients [14]. Patients with symptoms persisting beyond 6 months also demonstrated a clear sex difference, with a predominance of female patients. Additionally, the persistence of long COVID symptoms, particularly fatigue, for 6 months is a key criterion for meeting several diagnostic definitions of myalgic encephalomyelitis/chronic fatigue syndrome (ME/CFS) [15]. Consistent with this, we showed that 8.4% of patients with long COVID progressed to a clinical condition that fulfilled the diagnostic criteria for ME/CFS [15].

This mechanistic complexity underscores the need for a multidisciplinary rather than a monomodal treatment approach. Although several screening tools have been developed to aid symptom differentiation and elevated serum ferritin levels have been proposed as potential biomarkers for ME/CFS in patients with long COVID, no definitive biomarker has yet been established [8, 16–18]. Additionally, various endocrine factors, including the hypothalamic–pituitary axis, sex steroids, and thyroid hormones, may contribute to the onset and persistence of long COVID symptoms [19–23]. Our recent study reported that increased oxidative stress and decreased antioxidant capacity were observed in the post-COVID period, potentially contributing to the development of brain fog [13].

Measuring SARS-CoV-2 antibody titers is useful in providing clinically relevant information for long COVID management, even in the absence of recorded antigen testing during acute infection. However, the interpretation and significance of antibody titers in long COVID management remain unclear. Currently, the widespread availability of SARS-CoV-2 vaccines complicates serological surveillance. In the absence of additional information about the vaccination status of patients with long COVID, further tests capable of distinguishing between vaccination and natural infections are needed [24]. In this study, we aimed to determine whether serum antibody titers against SARS-CoV-2 spike (S) and nucleocapsid (N) proteins are useful for assessing the pathophysiology of patients with long COVID.

Because COVID-19 vaccines currently available in the United States and Japan target the S protein, S protein-based antibody tests reflect both vaccination and natural infection. In contrast, N protein-based tests reflect only natural infection. Given the widespread vaccination, the coexistence of vaccine-induced and infection-acquired immunity, and the occurrence of long COVID—even after unrecognized or mild Omicron infections—there is a need for clinical indicators that incorporate both S- and N-antibody titers. In the present study, we evaluated whether serum S- and N-antibody titers, which fluctuate during infection, could serve as biomarkers for managing patients with long COVID.

## Patients and Methods

### Study Design

This single-center retrospective cohort study was conducted at the COVID-19 Aftercare Clinic (CAC) of Okayama University Hospital. Data were retrospectively extracted from electronic medical records of patients who attended the CAC for their initial visit between 1 July 2023 and 30 November 2024.

### Inclusion Criteria for Patients With Long COVID

Patients were eligible for inclusion if they met the diagnostic criteria for long COVID at their initial CAC visit, had a confirmed history of COVID-19 onset, or were presumed to have been infected with the Omicron variant. Long COVID was defined as the persistence of symptoms for at least 30 days following COVID-19 onset [13]. Infection with the Omicron variant was defined as a SARS-CoV-2 infection occurring in the Okayama Prefecture on or after January 2022, according to regional epidemiological surveillance data [25]. Patients were excluded if the date of COVID-19 onset or vaccination history was unknown, or if at the initial visit, the attending physician determined that the primary reason for consultation was clearly attributable to a condition other than long COVID. Patients with a history of multiple SARS-CoV-2 infections were excluded.

### Collection of Clinical Data

Data were collected from the electronic medical records of patients attending the CAC at the Okayama University Hospital during the study period. Extracted variables included age, sex, smoking status, alcohol consumption history, body mass index (BMI), date of COVID-19 symptom onset, date of initial visit, severity of acute COVID-19, vaccination history, presenting symptoms, and laboratory findings. Questionnaire-based assessments included the FAS and SDS. The date of onset of COVID-19 symptoms was defined as the first day on which patients reported COVID-19-related symptoms. Duration from symptom onset to the initial CAC visit was calculated as the interval between the date of symptom onset and date of initial visit.

### Determination of Serum S- and N-Antibody Titers

Serum antibodies were assessed by each physician to evaluate past infection conditions and/or the possibility of vaccination. Serum samples obtained at initial visits were used for measurement of anti-SARS-CoV-2 spike (S)-protein (RBD) and anti-SARS-CoV-2 nucleocapsid (N)-protein using the Elecsys Anti-SARS-CoV-2 S RUO and Anti-SARS-CoV-2 RUO electrochemiluminescence (ECLIA) kits (Roche Diagnostics, Rotkreuz, Switzerland), respectively, under the operation with Cobas 8000 modular analyzer system at the Central Laboratory of Okayama University Hospital. For the analysis of Anti-SARS-CoV-2 S RUO, the measurement range is 0.40–25,000 U/mL (with 1:100 dilution) with a concentration of <0.80 U/mL considered negative. Threshold of the antibody titer was set to 200 U/mL to stratify patients based on a past report [26, 27]. For the analysis of Anti-SARS-CoV-2 RUO, the COI is 1.0, and values <1.0 indicate negative and ≥1.0 are considered positive. Antibody decay over time was evaluated using linear regression models with log-transformed antibody levels as the dependent variable and time from symptom onset to first visit as the independent variable. An exponential decay in antibody levels was assumed.

### Laboratory Examination

Blood samples were collected from each participant in a resting seated position during late morning outpatient visits. All specimens were routinely processed and analyzed using automated analyzers at the central laboratory of our hospital. Laboratory data on hemoglobin, inflammatory markers (CRP and ferritin), liver function parameters (albumin, aspartate aminotransferase, and alanine aminotransferase, renal function (creatinine), and low-density lipoprotein cholesterol were retrieved from the medical records and included in the analysis. Additionally, endocrine parameters, including adrenocorticotropin (ACTH), cortisol, free thyroxine (FT4), and thyrotropin (TSH) were measured [23]. Plasma ACTH and serum cortisol concentrations were measured using ECLIA with Elecsys ACTH and Elecsys Cortisol II assay kits (F. Hoffmann-La Roche AG), respectively. Serum FT4 and TSH levels were determined using Elecsys FT4 III and Elecsys TSH assay kits (F. Hoffmann-La Roche AG). Each laboratory parameter was measured once for each participant. All the analyses were conducted under rigorous quality control protocols in the hospital’s central laboratory, which is accredited by the Japan Accreditation Board in accordance with ISO 15189, which is the international standard for medical laboratory competence and quality management systems. Consequently, the reliability and reproducibility of laboratory results were consistently maintained in compliance with international standards.

### Evaluation of Patients’ QOL and Mental Status

The patients’ QOL and mental health status were evaluated at initial visit using standardized self-administered questionnaires. Fatigue severity was assessed using the FAS, a 10-item instrument comprising five items addressing physical fatigue and five addressing mental fatigue. Each item was scored on a 5-point Likert scale, yielding total scores between 10 and 50, with higher scores reflecting greater fatigue severity [28, 29]. Health-related quality of life was assessed using EuroQol 5-Dimension 5-Level (EQ-5D-5L), which evaluates five domains: mobility, self-care, usual activities, pain/discomfort, and anxiety/depression. Each domain is rated on a 5-level scale ranging from “no problems” to “unable to or extreme problems,” from which a summary QOL index score is derived [30]. Additionally, overall perceived health status was assessed using the EQ-5D VAS, with scores ranging from 0 (worst imaginable health state) to 100 (best imaginable health state). Depressive symptoms were evaluated using the SDS, a 20-item self-administered questionnaire in which each item is scored on a four-point scale. Higher total scores indicate greater severity of depressive symptoms [31].

### Statistical Analysis

All statistical analyses were performed using Stata/SE version 19 (StataCorp LLC, College Station, TX, USA) under an institutional license. Categorical variables are summarized as frequencies and percentages and compared using the Pearson’s chi-square test. Continuous variables are expressed as medians with IQRs and analyzed using the Mann–Whitney U test, as non-normal distributions were confirmed using the skewness–kurtosis test. Associations between the variables were examined using linear regression analysis and the Spearman’s rank correlation coefficients.

Because serum antibody titers were not normally distributed and showed a right-skewed distribution, log-transformed values were used in the multivariable regression analyses to improve model fit. Multivariable regression analyses were performed to evaluate factors associated with serum antibody titers and long COVID symptoms. Linear regression models were used for continuous outcomes (antibody titers), and logistic regression models were used for binary outcomes (symptoms). Covariates included age, sex, BMI, vaccination status, severity of acute COVID-19 infection, and the interval between COVID-19 onset and the initial clinic visit. For analyses examining associations between antibody titers and long COVID symptoms, antibody titers were included as explanatory variables in addition to these covariates. A two-sided *P* value of <0.05 was considered statistically significant.

Receiver operating characteristic (ROC) curve analyses were performed to evaluate the discriminatory performance of serum SARS-CoV-2 S-antibody titers for vaccination status and the presence of memory disturbance. The area under the curve (AUC) with 95% confidence intervals (CIs) was calculated to assess discriminative ability. Optimal cutoff values were determined by maximizing the Youden index, which maximizes the sum of sensitivity and specificity.

### Ethical Consideration

Information regarding the present study was made publicly available on the hospital website, and patients were provided with the opportunity to opt out of the study. As all patient data were fully anonymized, the requirement for informed consent was waived. This study was approved by the Ethics Committee of Okayama University Hospital (Approval No. 2105-030) and conducted in accordance with the principles of the Declaration of Helsinki.

## Results

**FIGURE 1:**
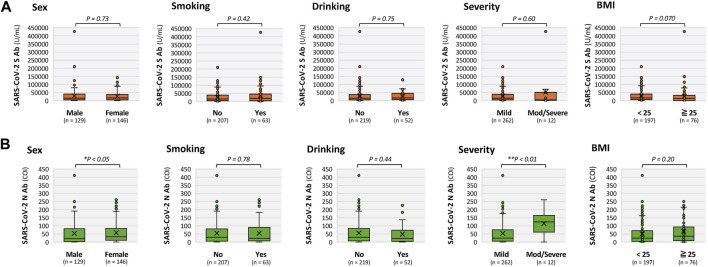
Comparison of SARS-CoV-2 spike (S) and nucleocapsid (N) antibody titers according to characteristics of patients with long COVID. Box-and-whisker plots comparing antibody titers according to gender, smoking status, alcohol consumption, acute COVID-19 severity (mild vs. moderate to severe), and body mass index (BMI <25 vs. ≥25 kg/m^2^). **(A)** S-antibody titers; and **(B)** N-antibody titers. S antibody-related plots are shown in orange, and N antibody-related plots are shown in green. Comparisons were performed using the Mann–Whitney U test. *P < 0.05 and **P < 0.01 indicate statistical significance. BMI: body mass index.

Baseline characteristics of 275 patients with long COVID (129 males, 46.9% and 146 females, 53.1%; median age, 41 years) are summarized in Table 1. Smoking history was more prevalent among male patients than that among females (29.1% vs. 18.2%, *P < 0.05), whereas alcohol consumption was not significantly different between the male and female patients. Median BMI was significantly higher in the males than that in the females (23.4 vs. 20.9, **P < 0.01; Table 1). Median serum spike (S) antibody titer was 14,879 U/mL in males and 17,084 U/mL in females, with no significant differences observed (*P =* 0.73; Table 1; Figure 1A). In contrast, median nucleocapsid (N) antibody titers were lower in males than those in females (20.6 vs. 33.0 cut-off index [COI], *P < 0.05; Table 1; Figure 1B). Median interval from the onset of COVID-19 symptoms to the initial clinic visit was significantly longer in males than that in females (197 vs. 123 days, *P < 0.05). Overall, 57 (20.7%) patients were unvaccinated (Table 1).

**TABLE 1:** Baseline characteristics of patients with long COVID during infection with the Omicron variant.

Baseline characteristics	Male (n = 129)	Female (n = 146)	*P* value
Age, median [IQR] Smoking habits, n (%) Alcohol habits, n (%) BMI, median [IQR]	39 [25–56] 37 (29.1; n = 127) 30 (23.4; n = 128) 23.4 [20.2–26.2; n = 128]	42.5 [28–54] 26 (18.2; n = 143) 22 (15.4; n = 143) 20.9 [18.7–24.8; n = 145]	0.41^(a)^ ^a^0.034^(b)^ 0.093^(b)^ ^b^0.003^(a)^
Serum SARS-CoV-2 S antibody (U/mL), median [IQR] Serum SARS-CoV-2 N antibody (COI), median [IQR]	14879 [4,966–41045] 20.6 [3.34–82.3]	17084 [4,222–38954] 33.0 [11.5–84.8]	0.73^(a)^ ^a^0.029^(a)^
Days from onset to the initial visit (days), median [IQR]	197 [73–408]	123 [63–296]	^a^0.023^(a)^
30–59 days, n (%) 60–89 days, n (%) 90–119 days, n (%) 120–149 days, n (%) 150 days and more, n (%)	22 (17.1) 16 (12.4) 13 (10.1) 4 (3.1) 74 (57.4)	31 (21.2) 24 (16.4) 17 (11.6) 11 (7.5) 63 (43.2)	​
Severity of acute condition, n (%)			
Mild (%) Moderate (%) Severe (%)	123 (95.4) 5 (3.9) 1 (0.8)	139 (95.9) 6 (4.1) 0 (0)	^a^0.96^(b)^
Vaccinations; n (%)			31 (21.2)
0 time (%) 1 time (%) 2 times (%) 3 times (%) 4 times (%) 5 times (%) 6 times (%) 7 times (%)	27 (20.9) 1 (0.8) 23 (17.8) 34 (26.4) 22 (17.1) 10 (7.8) 7 (5.4) 5 (3.9)	30 (20.5) 4 (2.7) 24 (16.4) 26 (17.8) 32 (21.9) 18 (12.3) 7 (4.8) 5 (3.4)	^##^0.94^(b)^

Data were analyzed using the (a) Mann–Whitney U or (b) chi-squared test.

^a^ *P* < 0.05 and.

^b^ *P* < 0.01 were regarded as statistically significant.

^c^ Mild vs. moderate/severe conditions.

^d^ 0 vs. 1- to 7-time vaccinations. COI, cut-off index; IQR, interquartile range.

Figure 1 also shows comparisons of SARS-CoV-2 S- and N-antibody titers according to sex, smoking status, alcohol consumption, severity of acute COVID-19 (mild vs. moderate to severe), and BMI (<25 vs. ≥25 kg/m^2^). For S-antibody titers (Figure 1A), no significant differences were observed according to smoking status [non-smokers: median = 15,487 (interquartile range, IQR: 4,222–39,237) vs. smokers: median = 18,779 (4,966–46,934), *P =* 0.42), alcohol consumption (non-drinkers: median = 15,594 (IQR: 4,389–38,954) vs. drinkers: median = 16,908 (5,281–45,136), *P =* 0.75], severity of acute COVID-19 [mild: median = 15,768 (IQR: 4,926–39,359) vs. moderate to severe: median = 5,066 (552–54,969), *P =* 0.60], or BMI [<25 kg/m^2^: median = 16,253 (IQR: 6,211–41,060) vs. ≥25: median = 13,693 (125–34,521), *P =* 0.070]. For N-antibody titers (Figure 1B), no significant differences were observed according to smoking status [non-smokers: median = 29.5 (IQR: 5.9–81.8) vs. smokers: median = 23.8 (6.9–92.3), *P =* 0.78], alcohol consumption [non-drinkers: median = 29.5 (IQR: 6.9–84.8) vs. drinkers: median = 22.5 (3.7425–79.325), *P =* 0.44], or BMI [<25 kg/m^2^: median = 24.6 (IQR, 4.975–73.3) vs. ≥25: median = 36.6 (9.0–96.0), *P =* 0.20]. In contrast, N-antibody titers were significantly higher in patients with moderate to severe acute COVID-19 than those in patients with mild disease [median = 124.5 (IQR: 44.6–166.0) vs. 25.1 (5.9–77.6), **P < 0.01].

**FIGURE 2:**
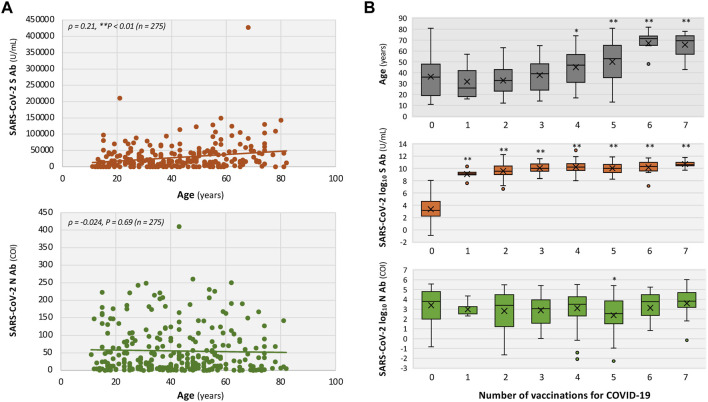
Associations of age and COVID-19 vaccination with SARS-CoV-2 spike (S) and nucleocapsid (N) antibody titers in patients with long COVID. **(A)** Scatter plots of age versus antibody titers for S (*upper*) and N (*lower*) antibodies. **(B)** Age and antibody titers according to the number of COVID-19 vaccine doses, with age shown in the *upper* plot and S- and N-antibody titers shown in the *middle* and *lower* plots, respectively. Associations were assessed using the Spearman’s rank correlation coefficient **(A)**; antibody titers in **(B)** are log-transformed and the comparisons vs. “0 vaccination group” were performed using the Mann–Whitney U test **(B)**. *P < 0.05 and **P < 0.01 indicate statistical significance.

As shown in Table 2, 57 (21%) of the 275 patients with long COVID were unvaccinated. The median S-antibody titers were significantly higher in the vaccinated patients than those in patients without vaccination (vaccinated vs. unvaccinated patients: 20,963 vs. 24 U/mL, **P < 0.01). Median N-antibody titer was 24.8 COI in the vaccinated patients and 44.5 COI in the unvaccinated patients, which were not significantly different (*P* = 0.072; Table 2). As shown in Figure 2A, serum S-antibody titers, but not N-antibody titers, were positively correlated with patients’ age (Spearman’s *ρ* = 0.21; **P < 0.01). Moreover, as shown in Figure 2B, patient age increased with the number of COVID-19 vaccine doses received. Median age was 36 years in the unvaccinated patients (n = 57), 26 years after one dose (n = 5), 33 years after two doses (n = 47), 39 years after three doses (n = 60), 47 years after four doses (n = 54), 53 years after five doses (n = 28), 71.5 years after six doses (n = 14), and 69.5 years after seven doses (n = 10). Patients who received four (*P < 0.05) or more (**P < 0.01) vaccine doses were significantly older than the unvaccinated patients (Figure 2B, *upper*).

**TABLE 2:** Serum titers of SARS-CoV-2 antibodies in patients with long COVID.

​	vaccinated (n = 218)	unvaccinated (n = 57)	*P* value
Serum SARS-CoV-2 S antibody (U/mL), median [IQR] Serum SARS-CoV-2 N antibody (COI), median [IQR]	20963 [11806–45807] 24.8 [5.3–70.5]	24 [8.5–111] 44.5 [7.4–122]	^a^<0.001 0.072

Data were analyzed using the Mann–Whitney U test.

^a^ *P* < 0.01 were statistically significant. COI, cut-off index; IQR, interquartile range.

**FIGURE 3:**
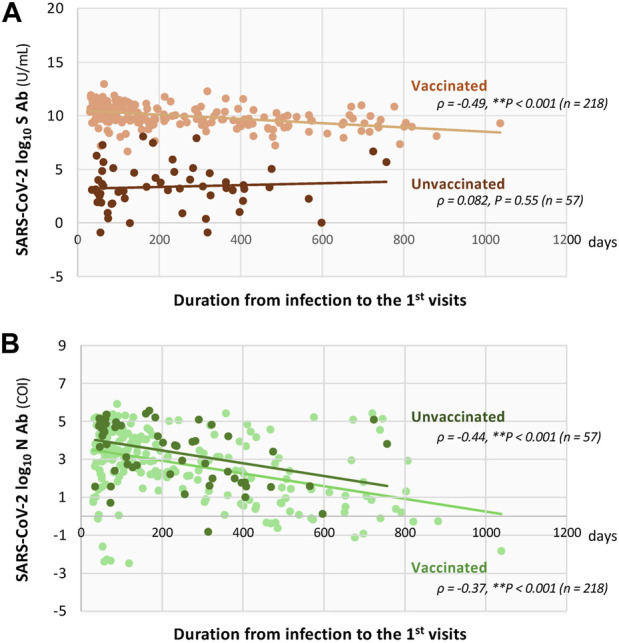
Time-dependent declines of serum titers of SARS-CoV-2 spike (S) and nucleocapsid (N) antibodies, stratified by vaccination status from COVID-19 onset to the first visit in patients with long COVID. Scatter plots showing antibody titers according to days from COVID-19 onset to the first visit. **(A)** The upper plot shows S-antibody titers, and **(B)** the lower plots shows N-antibody titers. Separate scatter plots were created for vaccinated and unvaccinated patients with long COVID and fitted trend curves are shown for each group. Associations were assessed using both the Pearson’s and Spearman’s rank correlation coefficients. *P < 0.05 and **P < 0.01 indicate statistical significance.

Serum S-antibody titers (Figure 2B, *middle*), but not N-antibody titers (Figure 2B, *lower*), were associated with the number of vaccine doses received. Median S-antibody titers increased in the unvaccinated patients from 24 U/mL to 9,215 U/mL after one dose, 13,937 U/mL after two doses, 22,594 U/mL after three doses, 27,056.5 U/mL after four doses, 22,096.5 U/mL after five doses, 31,465 U/mL after six doses, and 40,751 U/mL after seven doses (Figure 2B, *middle*). As shown in Figure 3A, in the unvaccinated patients with long COVID, no significant association was observed between time from symptom onset and S-antibody levels (Spearman’s *ρ* = 0.082, *P =* 0.55). In contrast, the N-antibody titers showed a significantly negative correlation with the time interval from COVID-19 symptom onset to the initial visit, with an estimated daily decline of 0.34% in the unvaccinated patients with long COVID (*ρ* = −0.44, **P < 0.01; Figure 3B).

In the multivariable linear regression analyses (Tables 3, 4), the determinants of serum S- and N-antibody titers differed substantially. Because antibody titers showed a skewed distribution, additional analyses were performed using log-transformed values, which demonstrated higher explanatory power than analyses using raw antibody values. Serum S-antibody levels were strongly associated with the number of vaccine doses (**P < 0.01) and were inversely associated with age (**P < 0.01; Table 3). In contrast, N-antibody titers were primarily associated with infection-related factors, including disease severity (**P < 0.01) and the interval from symptom onset (**P < 0.01; Table 4). Higher serum N-antibody levels were observed in patients with more severe disease, and N-antibody titers declined with increasing time after infection. The number of vaccine doses was not associated with serum N-antibody levels (*P* = 0.460).

**TABLE 3:** Multivariable analysis of background factors with log-transformed serum titers of SARS-CoV-2 S antibody in patients with long COVID.

Variables	β coefficient	95% CI	*P* value
Age (per year increase)	−0.028	−0.044–−0.012	^a^0.001
Female sex (/male)	−0.059	−0.552–0.434	0.815
BMI (per 1 kg/m^2^ increase)	−0.031	−0.085–0.022	0.252
Days from onset to the initial visit	−0.00044	−0.002–0.0006	0.450
Severity of acute condition	0.23	−0.590–1.055	0.579
Number of vaccine doses	1.24	1.101–1.378	^a^<0.001

Data were analyzed using multivariable linear regression with log-transformed serum S-antibody titers as the dependent variable, adjusted for age, sex, body mass index (BMI), days from onset to the initial visit, severity of acute illness, and number of vaccinations.

^a^ *P* < 0.01 were statistically significant. Model statistics: *R*2 = 0.564, Adjusted *R*2 = 0.554. CI, confidence interval.

**TABLE 4:** Multivariable analysis of background factors associated with log-transformed serum titers of SARS-CoV-2 N antibody in patients with long COVID.

Variables	β coefficient	95% CI	*P* value
Age (per year increase)	−0.014	−0.026–−0.002	^a^0.024
Female sex (/male)	0.489	0.112–0.866	^a^0.011
BMI (per 1 kg/m2 increase)	0.047	0.006–0.087	^a^0.025
Days from onset to the initial visit	−0.003	−0.004–−0.003	^b^<0.001
Severity of acute condition	1.082	0.453–1.710	^b^0.001
Number of vaccine doses	−0.040	−0.145–0.066	0.460

Data were analyzed using multivariable linear regression with log-transformed serum N-antibody titers as the dependent variable, adjusted for age, sex, body mass index (BMI), days from onset to the initial visit, severity of acute illness, and number of vaccinations.

^a^ *P* < 0.05 and.

^b^ *P* < 0.01 were statistically significant. Model statistics: *R*2 = 0.242, Adjusted *R*2 = 0.224. CI, confidence interval.

**FIGURE 4:**
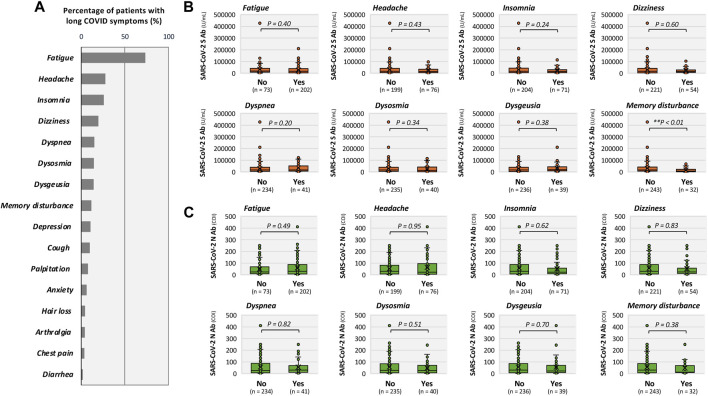
Serum titers of SARS-CoV-2 antibodies related with long COVID symptoms. **(A)** Distribution of major long COVID symptoms presented as horizontal bar graphs indicating the proportion of patients. **(B,C)** show box-and-whisker plots comparing antibody titers between patients with and without each symptom of long COVID, with S antibody shown in **(B)** and N antibody shown in **(C)**. Comparisons were performed using the Mann–Whitney U test; **P < 0.01 indicate statistical significance.

The percentage of patients with long COVID symptoms at presentation is shown in Figure 4A. Fatigue was the most common symptom reported by 73.5% of the patients, followed by headache (27.6%) and insomnia (25.8%). Among long COVID symptoms, S-antibody titers were significantly lower in patients with memory disturbance than those in patients without memory disturbance (median [IQR]: 6117 [25.0–23656] vs. 16029 [5974–41136] U/mL; **P < 0.01; Figure 4B). No significant differences in S-antibody titers were observed for other symptoms characterized by long COVID. Regarding N-antibody titers, there were no significant differences between patients with and without prolonged COVID symptoms (Figure 4C).

To further evaluate the association between serum S-antibody titers and long COVID symptoms, multivariable logistic regression analyses were performed (Table 5). Eight major long COVID symptoms—fatigue, headache, insomnia, dizziness, dyspnea, dysosmia, dysgeusia, and memory disturbance—were analyzed as dependent variables. Three models were evaluated: a crude model, Model I adjusted for age and sex, and Model II further adjusted for body mass index (BMI), days from infection, severity of acute illness, and vaccination status. In the fully adjusted Model II, higher S-antibody levels were significantly associated with a lower likelihood of memory disturbance (OR: 0.725, 95% CI: 0.542–0.971, *P < 0.05). No significant associations were observed between S-antibody titers and other symptoms, including fatigue, headache, insomnia, dizziness, dyspnea, dysosmia, or dysgeusia. The inverse association between S-antibody titers and memory disturbance was consistently observed across the crude and adjusted models (Table 5).

**TABLE 5:** Association between log-transformed serum S-antibody levels and long COVID symptoms based on multivariable logistic regression analysis.

Symptoms	Crude model		Model I		Model II	
	OR (95% CI)	*P* value	OR (95% CI)	*P* value	OR (95% CI)	*P* value
Fatigue	0.954 (0.867–1.048)	0.326	0.956 (0.868–1.052)	0.356	0.955 (0.759–1.201)	0.694
Headache	1.031 (0.941–1.129)	0.519	1.072 (0.972–1.183)	0.164	1.000 (0.794–1.259)	0.999
Insomnia	0.956 (0.876–1.042)	0.305	0.953 (0.872–1.041)	0.287	0.895 (0.719–1.114)	0.321
Dizziness	1.029 (0.928–1.142)	0.586	1.026 (0.923–1.140)	0.635	1.138 (0.881–1.470)	0.323
Dyspnea	1.042 (0.925–1.173)	0.498	1.010 (0.896–1.138)	0.873	0.932 (0.700–1.241)	0.632
Dysosmia	0.924 (0.835–1.023)	0.129	0.920 (0.829–1.021)	0.117	0.890 (0.674–1.175)	0.412
Dysgeusia	1.044 (0.924–1.179)	0.488	1.048 (0.925–1.187)	0.464	0.953 (0.700–1.299)	0.762
Memory disturbance	0.848 (0.764–0.941)	^a^0.002	0.841 (0.756–0.936)	^a^0.001	0.725 (0.542–0.971)	^b^0.031

Multivariable logistic regression analyses were conducted to evaluate associations between log-transformed serum S-antibody titers and eight major long COVID, symptoms—fatigue, headache, insomnia, dizziness, dyspnea, dysosmia, dysgeusia, and memory disturbance. Analyses were performed across three models: Crude model (n = 275), Model I adjusted for age and sex (n = 275), and Model II, fully adjusted for age, sex; BMI, days from infection, severity of acute illness, and vaccination status (n = 260–272), based on the Common Cause Approach. Odds ratios (ORs), 95% confidence intervals (CIs), and *P* values are shown for each model; and.

^a^ *P* < 0.01 were statistically significant.

^b^ *P* < 0.05 and.

**FIGURE 5:**
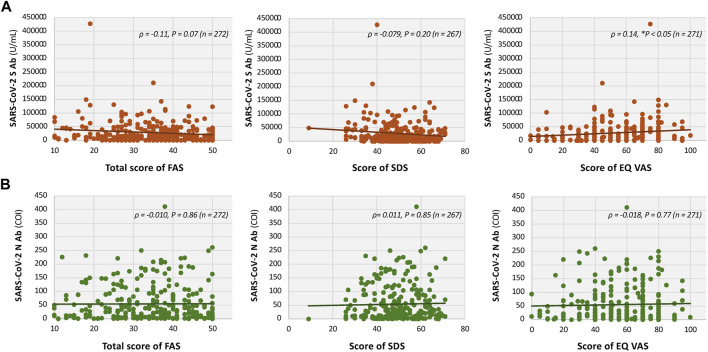
Interrelationships of fatigue, depression, and QOL scores with serum titers of SARS-CoV-2 antibodies in patients with long COVID. Scatter plots showing the relationships between Fatigue Assessment Scale (FAS) (*left panels*), Self-Rating Depression Scale (SDS) (*middle panels*), and EuroQol Visual Analogue Scale (EQ-VAS) (*right panels*) scores with antibody titers. **(A)** S-antibody titers, and **(B)** N-antibody titers. Associations were assessed using the Spearman’s rank correlation coefficient, with *ρ* and *P* values shown in the figure. *P < 0.05 and **P < 0.01 indicate statistical significance. QOL, quality of life.

Moreover, weak negative correlations were observed between S-antibody titers and fatigue score assessed by the Fatigue Assessment Scale (FAS), as well as depression score assessed by the Self-Rating Depression Scale (SDS; Figure 5). However, these correlations were not statistically significant (FAS: Spearman’s *ρ* = −0.11, *P =* 0.07, n = 272; SDS: *ρ* = −0.079, *P =* 0.20, n = 267; Figure 5A). No significant correlations were observed between the N-antibody titers and FAS or SDS scores (Figure 5B). EuroQol Visual Analogue Scale (EQ-VAS) scores significantly correlated with serum levels of S-antibody titer (*ρ* = 0.14, *P < 0.05, n = 271), but not with N-antibody titer (Figures 5A,B).

**FIGURE 6:**
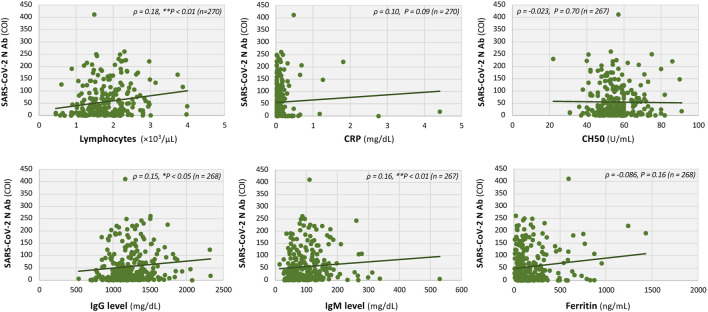
Interrelationships between serum laboratory markers and titers of SARS-CoV-2 N antibodies in patients with long COVID. Scatter plots showing the relationships between the levels of serum N-antibody titers and various laboratory parameters, including lymphocyte count, C-reactive protein (CRP), immunoglobulin G (IgG), immunoglobulin M (IgM), CH50, and ferritin. The associations were statistically analyzed using the Spearman’s rank correlation coefficient, with *ρ* and *P* values shown in the figure. *P < 0.05 and **P < 0.01 indicate statistical significance.

Finally, we analyzed the correlation between N-antibody titers and a wide range of clinical parameters in the blood of patients with long COVID, including immunological, hormonal, and nutritional markers (Figure 6). Results showed a positive correlation between N-antibody titers and lymphocyte counts (Spearman’s *ρ* = 0.18, **P < 0.01), immunoglobulin G (IgG) levels (*ρ* = 0.15, *P < 0.05), and immunoglobulin M (IgM) levels (*ρ* = 0.16, **P < 0.01). However, serum C-reactive protein (CRP) levels, complement level CH50, which has been reportedly associated with the brain fog symptoms of long COVID [32, 33], and serum ferritin, which has also been reported to be associated with ME/CFS symptoms [16, 17], did not show a significant correlation with serum N-antibody titers in patients with long-term COVID (Figure 6).

**FIGURE 7:**
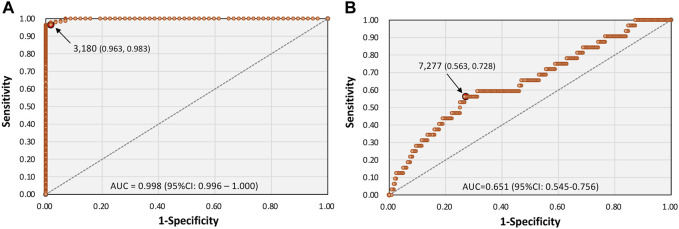
Receiver operating characteristic (ROC) curve analysis of serum SARS-CoV-2 S-antibody titers for vaccination status and memory disturbance. ROC curves were generated to assess the discriminatory ability of S-antibody titers for **(A)** vaccination status (no vaccination: 57 cases; past vaccination: 218 cases) and **(B)** memory disturbance in long COVID (yes: 32 cases; no: 243 cases). Optimal cut-off values were determined by maximizing the Youden index, with corresponding sensitivity and specificity indicated by red markers. The diagonal line represents random classification. AUC, area under the curve; CI, confidence interval.

Finally, the ROC curve analyses were performed to evaluate the discriminative ability of serum S-antibody titers (Figure 7). Serum S-antibody levels showed excellent discrimination for vaccination status (AUC = 0.998), with an optimal cutoff value of 3,180 U/mL (sensitivity 0.963, specificity 0.983). In contrast, the discriminative ability for memory disturbance was modest (AUC = 0.651) with an optimal cutoff value of approximately 7,277 U/mL (sensitivity 0.563, specificity 0.728), indicating that S-antibody levels alone have limited predictive value for cognitive symptoms.

## Discussion

Based on our retrospective observational study on the long COVID patients infected with the Omicron variant, it was revealed that S-antibody titers were associated with the number of vaccinations and specific long COVID symptoms, whereas during the acute phase of COVID-19, the N-antibody titers correlated with disease severity and were higher in women. N-antibody titers in unvaccinated patients with long COVID were negatively correlated with the time interval between infection and medical consultation, with an estimated daily decline in measured N-antibody levels of 0.34%. Interestingly, patients with memory impairment as a symptom of long COVID had low S-antibody titers, and a decline in S antibodies was associated with a decline in patient self-rated QOL measurement. These data suggest that measuring S- and N-antibody titers, even during the Omicron phase of infection, may provide insights into background characteristics associated with long COVID risk, and that a decrease in S-antibody titers may serve as a predictive indicator of long COVID risk.

Antibody responses to SARS-CoV-2 vary over time depending on the assay [24]. In all SARS-CoV antibody assays, disease severity consistently and strongly affects antibody titers in a dose-dependent manner [24]. Therefore, detection of past SARS-CoV-2 infection using antibody tests is highly dependent on the severity of initial infection, timing of specimen collection relative to infection, and testing method used [24]. Previous studies investigating the pre-Delta phase have shown that low SARS-CoV-2 antibody titers during the acute phase may be a risk factor for prolonged symptoms [34, 35]. However, detailed studies are required to determine the usefulness of antibody titers in predicting long-term COVID symptoms during the Omicron-dominant phase in current vaccination settings.

Current COVID-19 antibody tests use various SARS-CoV-2 antigen targets, including spike protein (S), nucleocapsid protein (NCP), receptor-binding domain (RBD), and immunoglobulins [36–38]. The NCP is involved in RNA packaging and viral particle release, and the transmembrane spike glycoprotein consists of two functional subunits: the N-terminal S1 subunit, which binds to host cell receptors, and the C-terminal S2 subunit, which fuses the viral membrane with the cellular membrane [39]. The RBD is located in the C-terminal region of the S1 subunit [40]. The RBD interacts with angiotensin-converting enzyme 2 (ACE2) expressed on human cells, thereby mediating viral entry. Antibody responses against SARS-CoV-2 infection are directed against multiple viral antigens, including diverse epitopes on the spike protein. In particular, antibodies targeting the RBD located in the C-terminal region of the S1 subunit, measured as S antibodies in the present study, are considered to exert neutralizing activity [41].

Regarding anti-RBD antibodies, we previously reported the characteristics of serum SARS-CoV-2 S antibody titers in patients with long COVID infected during the pre-Delta phase before July 2021 [42]. In our previous study, serum S-antibody titers were elevated in patients who experienced severe acute phase; however, they did not appear to be associated with persistent symptoms of long COVID. Patients with S-antibody titers >200 U/mL at their initial visit exhibited a significantly greater number of persistent symptoms; however, this difference in residual symptoms had resolved by the third month, corresponding to the onset period of long COVID [42]. These results suggest that measuring serum S-antibody titers in patients with long COVID is not a suitable indicator of persistence of long-term symptoms in patients infected with COVID-19 during the pre-Delta phase [42].

Furthermore, previous studies have demonstrated that serological response kinetic models show antibody responses that depend on the number of days since symptom onset and illness severity [37]. A rapid increase in antibody titers was observed depending on the test method and target population, followed by a plateau or decline. In hospitalized patients, using the same N-antibody test method as in this study, the overall maximum responses peaked at to10 months, and the antibody responses were more sustained [37]. Given our observation that N-antibody titers in unvaccinated patients with long COVID declined at an estimated rate of 0.34% per day and were negatively correlated with the interval between infection and medical consultation, the measurement of N antibodies against NCP may be a useful tool for inferring prior COVID-19 infection and its timing, especially in contemporary clinical practice where antigen or polymerase chain reaction testing is increasingly omitted.

Female sex has been consistently identified as a risk factor for long COVID in multiple previous studies [43–46]. We previously reported that women account for majority of the patients with long COVID (59.4%), and that long COVID can develop even in women with only mild acute symptoms [14]. Additionally, we have shown that long COVID is more prevalent among women in their 40s, and that menstrual irregularities accompanied by depressive symptoms became more common following infection during the Omicron-dominant phase [20]. In the present study, serum N-antibody titers correlated with severity of the acute phase of COVID-19 and were significantly higher in female patients than those in male patients with long COVID. The increased risk of long COVID in women is likely affected by a combination of biological and social factors [47]. Biologically, a decrease in estrogen levels may contribute to immune abnormalities after COVID-19 infection. Furthermore, given the observed worsening mental health and lifestyle changes in women [5, 48]. The COVID-19 pandemic itself may have exacerbated gender disparities [49, 50].

Overall, the present study demonstrates that the determinants of SARS-CoV-2 antibody responses differ depending on the target antigen in long COVID patients. Serum S-antibody levels were primarily determined by vaccination history, whereas N-antibody levels reflected infection-related factors such as disease severity and time since infection. These findings highlight the distinct immunological mechanisms underlying vaccine-induced and infection-induced humoral responses in patients with long COVID. Notably, age was inversely associated with both S- and N-antibody titers, possibly reflecting age-related attenuation of humoral immune responses implying immunosenescence condition [51]. In addition, the inverse association between N-antibody titers and time from symptom onset may reflect the waning of infection-induced humoral immunity over time. These findings may help improve the interpretation of serological profiles in patients with long COVID and contribute to a better understanding of the immunological background of persistent post-COVID conditions.

A prospective study in Poland investigated whether adaptive humoral anti-SARS-CoV-2 responses differed in patients with long COVID [52]. The enrolled adult patients were infected in Poland before the Delta-variant phase, and their clinical outcomes were similar [53, 54]. The patients in this study had not been vaccinated against COVID-19 and had no history of reinfection. The diagnosis of long COVID is based on persistent symptoms, and clinical phenotypes are classified into three categories: cardiac, pulmonary, and psychiatric. Interestingly, all the three phenotypes showed significantly reduced seropositivity for anti-NCP IgG antibodies, suggesting that long COVID is associated with reduced odds of N-antibody positivity. Furthermore, seropositive patients with long COVID had lower levels of anti-S1 and anti-S2 antibodies than patients with non-long COVID, and patients with long COVID having pulmonary and psychiatric phenotypes also had reduced levels of anti-RBD antibodies, which are synonymous with our S antibodies [52]. These results suggest that long COVID is characterized by reduced humoral immunity against SARS-CoV-2.

In our current study, serum S-antibody titers were not significantly associated with most long COVID symptoms, including fatigue, headache, insomnia, dizziness, dyspnea, dysosmia, and dysgeusia. However, higher S-antibody levels were associated with a lower likelihood of memory disturbance even after multivariable adjustment. Notably, this association remained significant and became more pronounced after adjustment for demographic and clinical covariates, suggesting that the relationship was not explained by major confounding factors. In addition, the ROC analysis showed that serum S-antibody titers performed well in discriminating vaccination status and reflected a strong immune response to spike vaccines. But their ability to discriminate memory impairment was insufficient, suggesting that antibody titers alone are insufficient as a definitive clinical biomarker for predicting cognitive symptoms caused by long COVID.

The present study has several limitations. Because the study population consisted of patients who visited specialized outpatient clinics at university hospitals, it was not possible to eliminate selection bias towards more severe or treatment-resistant cases. Further studies involving a larger number of patients through more nationwide, multicenter collaborative research are needed. Furthermore, this study is a retrospective observational study and does not comprehensively evaluate all potential confounding factors such as pre-existing conditions and comorbidities. Also, the discriminatory performance of ROC analysis and the findings from multivariate models should be interpreted with caution due to sample-specific cut-off values, potential residual confounding, multicollinearity, and the assumptions of linear relationships, which may limit generalizability.

In conclusion, determinants of SARS-CoV-2 antibody responses differed according to the target antigen in patients with long COVID even in the absence of antigen test records during acute infection, suggesting the measurement of SARS-CoV-2 antibody titers provides clinically important information for long COVID management. Serum N-antibody titers reflect past SARS-CoV-2 infection, disease severity, and immune responses. However, it should be noted that N-antibody titers tend to be higher in women, are affected by the interval between infection and evaluation, and decline at an estimated rate of 0.34% per day. In contrast, although S-antibody titers largely reflect vaccination status, a decrease in S-antibody titers may be associated with brain fog symptoms, such as memory impairment, related to the patients’ QOL. Therefore, although antibody profiles characterized by “N-antibody positivity and decreased S-antibody titers” are useful in the clinical setting of long COVID, careful consideration of various background factors is essential for their interpretation. Further studies are warranted to clarify the clinical implications of these findings.

## Summary Table

### What Is Known About This Topic

- Long COVID causes persistent symptoms, but reliable biomarkers reflecting clinical features remain limited.
- SARS-CoV-2 S-antibodies mainly reflect vaccination, whereas N-antibodies indicate natural infection.
- Host immune responses may contribute to heterogeneity of long COVID symptoms after Omicron infection.

### What This Work Adds

- In Omicron-related long COVID, N-antibody titers reflected immune responses and acute disease severity.
- Lower S-antibody titers were independently associated with brain fog and reduced quality of life.
- Antibody profiles varied with vaccination history, sex, and time since infection.

## Final Summary Sentence

This work represents an advance in biomedical science because differential patterns of S- and N-antibody titers may serve as potential immune biomarkers reflecting clinical manifestations in Omicron-related long COVID.

## Data Availability

The raw data supporting the conclusions of this article will be made available by the authors, without undue reservation.
